# Characterization of natural polymorphic sites of the HIV-1 integrase before the introduction of HIV-1 integrase inhibitors in Germany

**DOI:** 10.7448/IAS.17.4.19746

**Published:** 2014-11-02

**Authors:** Karolin Meixenberger, Kaveh Pouran Yousef, Sybille Somogyi, Stefan Fiedler, Barbara Bartmeyer, Max von Kleist, Claudia Kücherer

**Affiliations:** 1 HIV and Other Retroviruses, Robert Koch Institute, Berlin, Germany; 2 Department of Mathematics and Computer Science, Freie Universität Berlin, Berlin, Germany; 3 Nosocomial Pathogens and Antibiotic Resistances, Robert Koch Institute, Berlin, Germany; 4 HIV/AIDS, STI and Blood-borne Infections, Robert Koch Institute, Berlin, Germany

## Abstract

**Introduction:**

The aim of our study was to analyze the occurrence and evolution of HIV-1 integrase polymorphisms during the HIV-1 epidemic in Germany prior to the introduction of the first integrase inhibitor raltegravir in 2007.

**Materials and Methods:**

Plasma samples from drug-naïve HIV-1 infected individuals newly diagnosed between 1986 and 2006 were used to determine PCR-based population sequences of the HIV-1 integrase (amino acids 1–278). The HIV-1 subtype was determined using the REGA HIV-1 subtyping tool. We calculated the frequency of amino acids at each position of the HIV-1 integrase in 337 subtype B strains for the time periods 1986–1989, 1991–1994, 1995–1998, 1999–2002, and 2003–2006. Positions were defined as polymorphic if amino acid variation was >1% in any period. Logistic regression was used to identify trends in amino acid variation over time. Resistance-associated mutations were identified according to the IAS 2013 list and the HIVdb, ANRS and GRADE algorithms.

**Results:**

Overall, 56.8% (158/278) amino acid positions were polymorphic and 15.8% (25/158) of these positions exhibited a significant trend in amino acid variation over time. Proportionately, most polymorphic positions (63.3%, 31/49) were detected in the N-terminal zinc finger domain of the HIV-1 integrase. Motifs and residues essential for HIV-1 integrase activity were little polymorphic, but within the minimal non-specific DNA binding region I220-D270 up to 18.1% amino acid variation was noticed, including four positions with significant amino acid variation over time (S230, D232, D256, A265). No major resistance mutations were identified, and minor resistance mutations were rarely observed without trend over time. E157Q considered by HIVdb, ANRS, and GRADE algorithms was the most frequent resistance-associated polymorphism with an overall prevalence of 2.4%.

**Conclusions:**

Detailed knowledge of the evolutionary variation of HIV-1 integrase polymorphisms is important to understand the development of resistance in the presence of the drug. Our results will contribute to define the relevance of integrase polymorphisms in HIV-strains resistant to integrase inhibitors and to improve resistance interpretation algorithms.

